# Significant Detection of Porcine Circovirus 3 and Porcine Circovirus 4 in Wild Boars from Mid-Western Spain Without Apparent Sanitary Consequences

**DOI:** 10.3390/ani15040523

**Published:** 2025-02-12

**Authors:** Rocío Holgado-Martín, David Risco, Alfonso Ramos, Remigio Martínez-Pérez, Waldo Luis García-Jiménez, Javier Hermoso-De Mendoza, Luis Gómez

**Affiliations:** 1 Departamento de Medicina Animal, Unidad de Anatomía Patológica y Anatomía Comparada, Facultad de Veterinaria de Cáceres, Universidad de Extremadura, 10003 Cáceres, Spain; rociohm@unex.es (R.H.-M.); walgarjim@unex.es (W.L.G.-J.); luih@unex.es (L.G.); 2 Departamento de Matemáticas, Unidad de Estadística e Investigación Operativa, Facultad de Veterinaria de Cáceres, Universidad de Extremadura, 10003 Cáceres, Spain; aramos@unex.es; 3 Grupo de Investigación en Sanidad Animal y Zoonosis (GISAZ), Departamento de Sanidad Animal, UIC Zoonosis y Enfermedades Emergentes ENZOEM, Facultad de Veterinaria de Córdoba, Universidad de Córdoba (ROR code 05yc77b46), 14014 Córdoba, Spain; remimar@unex.es; 4 Departamento de Salud Animal, Unidad Patología Infecciosa, Facultad de Veterinaria de Cáceres, Universidad de Extremadura, 10003 Cáceres, Spain; jhermoso@unex.es

**Keywords:** porcine circovirus 3, porcine circovirus 4, epidemiology, wild boars

## Abstract

PCV-3 and PCV-4 are novel viruses that can infect domestic pigs and wild boars. Both viruses are associated with multiple disorders in domestic pigs (reproductive failure, respiratory damage, etc.). However, the clinical impact on wild boars remains unknown. This study aimed to assess the presence of these viruses in wild boars from mid-western Spain and their sanitary impact on the species. A total of 166 submandibular lymph nodes were collected from hunted wild boars, along with available information about their reproductive status, lung injuries, body condition, and tuberculosis status. The samples were used to detect PCV-3 and PCV-4 using real-time PCR. In total, 84.9% of the sampled animals were positive for PCV-3, and 33.7% were positive for PCV-4. The detection of PCV-4 was more frequent in wild boars that had received supplementary feeding, suggesting that the direct contact favored by this practice could increase the transmission of this virus in wild boar populations. The infections did not seem to influence the body condition, reproductive status, lung lesions, or TB lesion severity patterns in the studied animals. Thus, although these viruses have been widely detected throughout wild boar populations from the studied area, they do not seem to be a health threat to this species. Nevertheless, their monitoring in wild boars is recommended, as they are often in contact with extensively reared pigs, which are susceptible to these viruses.

## Simple Summary

Porcine circoviruses 3 and 4 (PCV-3 and PCV-4) are recently discovered viruses that can infect both domestic pigs and wild boars. The presence of these viruses in wild boars from mid-western Spain was assessed using submandibular lymph nodes collected during routine hunting inspections. In total, 84.9% of the animals were positive for PCV-3, and 33.7% were positive for PCV-4, with PCV-3 showing higher positivity rates than those reported in previous studies carried out in Spain. A total of 38.3% of PCV-4-positive wild boars received supplementary feeding, suggesting that the direct contact that occurs during this practice could increase the transmission of this virus. Lymph nodes appear to be more effective for detecting these viruses than other kinds of samples. Although no severe disease symptoms were observed in the wild boars, monitoring these viruses is recommended, as wild boars are often in contact with extensively reared pigs, which are more susceptible to these viruses.

## 1. Introduction

Disease emergence is a worldwide continuous process in which a major inconvenience is the frequent absence of measures for prevention or control [1]. In the case of porcine circoviruses (PCVs), two new types have been found in the last few years, namely, porcine circovirus 3 (PCV-3) and porcine circovirus 4 (PCV-4), being assigned consecutive denominations to PCV-1 and PCV-2. PCVs belong to the *Circoviridae* family, which comprises small icosahedral and non-enveloped viruses with single-stranded and circular DNA genomes. PCVs are the smallest viruses known to infect animals [2,3]. PCV-2 is considered the most pathogenic so far, generating considerable economic losses in the porcine industry worldwide [4,5], but little is known about the most recent PCVs.

PCV-3 was identified in 2015 in the USA in PCV-2-negative sows displaying signs of Porcine Dermatitis and Nephropathy Syndrome (PDNS) [6]. Subsequently, PCV-3 was detected in South America, Europe, and Asia and is now considered almost ubiquitous [7]. Despite its recent discovery, retrospective studies have demonstrated its circulation since at least 1993 in Sweden [8] and 1996 in Spain [9] and China [10]. In Italy, besides detecting PCV-3 in a high proportion of wild boars, it has been observed to have low prevalence in other wild ungulates such as chamois (*Rupicapra rupicapra*) and roe deer (*Caprelous caprelous*). This could indicate a certain plasticity of the virus to host adaption [11].

PCV-3 affects the commercial porcine sector and has also been detected in wild boar (*Sus scrofa*) populations [12]. Besides showing signs of PDNS, PCV-3 infections have been related to reproductive disorders, respiratory symptoms, and myocarditis in domestic pigs [6,13,14,15], and they have also been detected in asymptomatic animals [16,17].

PCV-3 viral antigens have been detected in multiple tissues and organs such as the lungs, liver, spleen, kidneys, heart, lymph nodes, and intestines [18]. Furthermore, PCV-3 has been identified in lymphoid organs, including the lymph nodes and spleen, and it has been observed in the lymphocytes, macrophages, neutrophils, eosinophils, and epithelial cells of infected tissues [6,18]. Surprisingly, it has also been detected in fecal samples, suggesting a fecal–oral transmission route [19]. A study focused on PCV-3 detection found the highest percentage of positivity in submandibular lymph nodes, followed by the tonsils, lungs, liver, spleen, and kidneys, with serum samples showing the lowest rates. Long-term infection in individuals was also studied, verifying cases of reinfections or persistent infections [9].

Meanwhile, PCV-4 was discovered in 2019 in domestic pigs in China [20]. Unlike PCV-3, the newly discovered PCV-4 has only been described in specific areas to date. Despite its recent identification, retrospective studies have demonstrated infections since at least 2008 [21,22]. Outside of China, the PCV-4 genome was subsequently found in Korea, Thailand, Spain, Malaysia, Mongolia, the United States, and Taiwan [23,24,25,26,27,28,29]. Although several studies attempted to identify it in other countries, such as Italy and Colombia, no evidence of this virus was found [30,31]. After its detection in pigs in China, PCV-4 was subsequently detected in wild boars [32]. Its DNA has been detected in two other non-swine domestic species, namely, dairy cows and dogs from China [33,34]. This virus has been found in a variety of tissues from pigs, including the heart, liver, spleen, lungs, kidneys, lymph nodes, intestines, and brain [22]. PCV-4 DNA has been detected in domestic pigs affected by respiratory disorders, PDNS, postweaning multisystemic wasting syndrome (PMWS), neurological issues, diarrhea, enteritis, encephalitis, respiratory disease, and reproductive disorders [20,22,27,28,29,35,36]. Although the symptomatology and pathogenesis of PCV-4 appear to be similar to those in PCV-2 infection, a connection has not been clearly established, and the symptomatology of PCV-4 infection is still not well defined, as the virus has also been found in apparently healthy animals [20].

Since PCV-3 and PCV-4 have been detected in wild boars, these pathogens may pose a sanitary threat to this species, as boars are susceptible to circoviral infections, mainly in areas with a high population density [37]. It has been shown that PCV-2-infected wild boars can suffer from similar symptoms to pigs [37], leading to patent disease [5]. Furthermore, PCV-2 has been shown to have strong immunosuppressive effects, with its infection even being linked to an increased severity of other key diseases affecting wild boar, such as tuberculosis (TB) [38]. However, the pathological implications and the immunosuppressive roles of PCV-3 and PCV-4 infections in wild boars are still unknown. Thus, the aims of this retrospective study were (1) to evaluate the existence of PCV-3 and PCV-4 in wild boar populations from mid-western Spain, analyzing the possible epidemiological factors that could favor the existence of infections by these viruses, and (2) to assess whether PCV-3 and PCV-4 infections in wild boars may affect their body condition, reproductive parameters, lung pathology, and the severity of TB lesions.

## 2. Materials and Methods

### 2.1. Animals and Area of Study

This study was based on a retrospective analysis of a set of samples and data obtained from 1518 wild boars, hunted and routinely inspected between 2011 and 2015 in different hunting events taking place on a total of 37 game estates located in mid-western Spain. This area has a predominant continental Mediterranean climate, with hot, dry summers and mild and moderately wet winters. The vegetation mainly consists of scrublands of *Cistus* spp., *Arbutus unedo*, and evergreen oak forests (*Quercus ilex* and *Quercus suber*). The wild boars on the different hunting estates usually share the habitat with other ungulates such as red deer (*Cervus elaphus*), roe deer (*Capreolus capreolus*), fallow deer (*Dama dama*), and European mouflon (*Ovis orientalis musimon*). The hunting estates sampled ranged between 380 and 2500 Ha and had different typologies, such as fenced, unfenced, and with or without supplementary feeding. Supplementary feeding involves the supply of animal food in the field for wild boars at different times of the year, with the goal of improving the quality of hunting trophies, reducing damage to natural plant cover, and attracting animals to hunting areas [37,39].

From the total number of sampled animals included in the initial dataset, we selected a subset of wild boars according to different criteria. Firstly, to make PCV-3 and PCV-4 diagnoses possible, we exclusively chose wild boars whose submandibular lymph nodes were stored at −20 °C. Secondly, among the previously selected animals, we chose those with known registered individual information (sex, age, body condition, nutrition), reproductive performance, evaluation of lung damage, and the presence of TB lesions, obtaining a final subset of 166 wild boars that were used for this study.

### 2.2. Sampling Procedures and Data Collection

**Figure 1:**
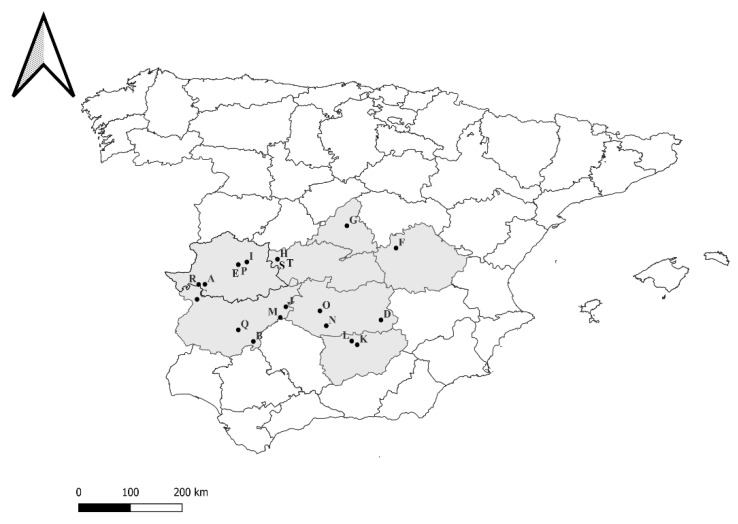
Locations (A–T) of the game estates whose wild boar were included in this study.

The hunted wild boars included in this study (*n* = 166) were characterized and sampled during different hunting events that took place between October 2011 and February 2015 on 20 game estates from seven provinces in mid-western Spain (Figure 1), following a similar methodology. During the outdoor veterinary inspection of carcasses after the hunts, 10 wild boars were randomly selected, marked with plastic tags, and sampled. In hunting events where fewer than 10 wild boars were hunted, all boars were sampled. Individual information such as sex (male or female) or age (<1 year = juvenile, 1–2 years = subadult, and >2 years = adult) was recorded by observing the genital organs and tooth eruption pattern, respectively [40,41]. Furthermore, the total length and chest perimeter were measured using a metric tape to predict their thoracic fat thickness following previously described protocols [42]; this parameter was used as a proxy for body condition [43]. Subsequently, a pathological examination of these animals was carried out, involving the inspection and sampling of organs (lung, submandibular lymph nodes, and reproductive tracts) to obtain information regarding their reproductive performance and the existence of lung damage and tuberculosis-like lesions.

### 2.3. PCV-3 and PCV-4 Diagnosis

Submandibular lymph nodes from 166 selected wild boars were used to assess the presence of PCV-3 and PCV-4 using molecular techniques. Briefly, DNA was extracted from lymph node tissues using an isolation kit (Nukex Mag RNA/DNA, Gerbion, Kornwestheim, Germany), following the manufacturer’s instructions, with the KingFisher™ Flex Purification System (Thermo Fisher Scientific, Waltham, MA, USA). Afterwards, the presence of these PCVs was evaluated with real-time PCR, using the RealQ Plus 2x Master Mix for Probe polymerase (AMPLIQON, Odense, Denmark). To detect PCV-3 DNA, final concentrations of 1.2 µM of primers (PCV-3_353_F and PCV-3_465_R) and 0.6 µM of probe (PCV-3_418_probe) were used; for the analysis of PCV-4, the final concentrations used were 0.6 µM of primers (PCV-4DF and PCV-4DR) and 0.3 µM of probe (PCV-4-prob) [20,44]. The primers used were specific for a fragment of the *Rep* gene of PCV-3 as well as of PCV-4, both with 193 bp.

### 2.4. Assessing Reproductive Parameters

The reproductive status of wild boar females was determined by inspecting their reproductive organs (ovaries, uterus, and breast). The females were categorized as breeding (when embryos/fetuses were found in the uterus or distended udders were observed during necropsy) or not breeding (when none of these features were detected) [45]. Furthermore, in pregnant females, the number of corpora lutea found in both ovaries and the number of fetuses were counted to calculate their potential fertility (litter size) and their intrauterine mortality (number of corpora lutea minus number of fetuses/number of corpora lutea × 100) [46,47].

### 2.5. Evaluation of Lung Damage

The presence of lung lesions was assessed through a microscopic examination of the lungs from hunted wild boars, following previously described protocols [48]. Briefly, a detailed macroscopic inspection of the lungs was performed during the outdoor field necropsies. In each case, a sample from the right cranial lung lobe was taken and immersed in 10% buffered formalin immediately after the examination and was processed and stained with hematoxylin and eosin for the subsequent histopathological study. Five histopathological parameters (presence of alveolar septal infiltration with inflammatory cells, amount of exudates in alveoli and airways, peri bronchial lymphoid hyperplasia, amount of inflammatory cells in the lamina propria of bronchi, and bronchioles and presence of necrosis in epithelial cells of bronchi and bronchioles) were scored on a scale from 0 to 6 (0, normal; 1, mild multifocal; 2, mild diffuse; 3, moderate multifocal; 4, moderate diffuse; 5, severe multifocal; 6, severe diffuse) [49,50]. The sum of all scores obtained for these five parameters (ranging between 0 and 30) was used as a proxy of total lung damage. Finally, the existence of vasculitis and perivasculitis was evaluated in a representative set of lung samples (*n =* 41).

### 2.6. TB Lesion Severity Assessment

During the necropsies, the main lymph nodes (submandibular, retropharyngeal, tracheobronchial, mediastinal, gastro-hepatic, and mesenteric) and thoracic and abdominal cavities and organs (lungs, liver, and spleen) were carefully examined to assess the presence of tuberculosis-like lesions (TBLLs). The animals displaying TBLLs were categorized according to the severity of TB as showing a “localized” pattern if lesions affected only cephalic or mesenteric lymph nodes, and a “generalized” pattern if they affected at least two different organs or lymph nodes from different regions [51].

### 2.7. Statistical Analysis

The results were analyzed using SPSS version 29 software (IBM, Chicago, IL, USA) to assess the presence of risk factors (sex, age, food supplementation) predisposing the wild boars to infection with PCV-3 and/or PCV-4 and the effect of these infections on generic or health parameters (body condition, reproductive performance, lung damage, or TB severity). The normality of the data was evaluated using the Shapiro–Wilk test. For comparisons between two groups, Student’s *t*-test was used for parametric data, while the Wilcoxon test was applied for non-parametric data. A 95% confidence interval (CI) was used in all analyses. Additionally, Chi-square and Fisher exact tests were used to compare the categorical variables among groups.

## 3. Results

### 3.1. PCV-3 and PCV-4 Detection Using PCR and Risk Factor Association

A total of 141 wild boars (84.9%) were found to be positive for PCV-3 DNA (mean Ct value 29.42 [19.9–39.5]), whereas PCV-4 DNA was detected in 56 wild boars (33.7%) (mean Ct value 34 [21.2–38.9]). PCV-3 and PCV-4 positivity was detected in samples obtained from the seven provinces in Spain where the hunting events were carried out, with different positivity rates (Table 1). Furthermore, the coexistence of PCV-3 and PCV-4 DNA was detected in 50 animals (30.12%).

No differences in PCV-3 and PCV-4 positivity rates were detected between male and female animals selected for this study, nor among different age groups (young, subadult, and adult) (see Table 2). PCV-4 positivity was higher in wild boars from estates that used food supplementation (38.3%) compared to those that did not (15.6%) (X = 4.86, *p* = 0.027). In contrast, no differences were detected regarding PCV-3 positivity between supplemented and non-supplemented animals (88% vs. 75%) (X = 3.1, *p* = 0.21).

### 3.2. Influence of PCV-3 and PCV-4 PCR Positivity on Health Parameters

The values obtained for the reproductive parameters and body condition in animals positive and negative for PCV-3 and PCV-4 are summarized in Table 3. Here, 63.9% (53/83) of the females studied were breeding, showing a mean potential fertility of 3.61 piglets and 16.84% of intrauterine mortality. When these females were clustered according to positivity for PCV-3 or PCV-4, no differences regarding any reproductive parameter were detected between positive and negative animals (Table 3). Conversely, the mean body condition was significantly higher in PCV-3-positive animals (17.6 mm) when compared to PCV-3-negative animals (14.3 mm) (Student’s *t*-test = −3.023, *p* = 0.004).

Regarding lung damage, the mean scores obtained for the histopathological parameters evaluated (0 to 6) and mean total lung damage score (0 to 30) are summarized in Table 4. None of these parameters showed significant differences when the population was clustered in animals positive or negative for PCV-3 and PCV-4 (Table 4). Furthermore, vasculitis with the presence of mild perivascular edema and several mononuclear cells (mainly lymphocytes) surrounding the affected vessels was detected in 14.63% (6/41) of the samples examined for this purpose. The percentage of samples displaying vasculitis was not influenced by positivity for PCV-3 or PCV-4. Similarly, no differences in the rate of animals displaying generalized TB lesions were registered between groups positive and negative for PCV-3 or PCV-4. The scores obtained for all of the histological parameters for the studied samples are fully available in Supplementary Table S2.

A total of 74 out of 139 wild boars in which a complete TB assessment could be carried out showed TBLLs. Of these, 29 (39.2%) displayed generalized TBLLs, whereas localized TBLLs were detected in 45 animals (60.8%). Among the wild boars positive for PCV-3 and PCV-4 according to PCR, 36.7% (22/60) and 52.2% (12/23) of TB-affected animals showed a generalized pattern, respectively, whereas 50% (7/14) and 33.3% (17/51) displayed localized TB lesions.

The values obtained for all of these health parameters (body condition, breeding status, potential fertility, intrauterine mortality, lung damage scores, and TB severity) were also compared between animals coinfected with PCV-3 and PCV-4 (*n* = 50) and those negative for both viruses (non-infected animals) (*n* = 19), but no significant differences were found regarding any of the studied variables.

## 4. Discussion

The results obtained in this study confirm the considerable presence of PCV-3 and PCV-4 in wild boar populations from mid-western Spain. The rate of detection of PCV-3 (84.9%) obtained in this area was higher than those registered in other surveys carried out in pigs and wild boars from Spain (42.7% in wild boars and 11.4% pigs) and Italy (61.5% in wild boars and 20% of domestic pigs) [9,52,53]. This remarkable difference could be due to the type of samples used, since we used submandibular lymph node tissues for the diagnosis, while in other studies, serum samples were used. The use of lymph node tissues as a target to detect PCV-3 and PCV-4 appears to be more efficient than other types of substrate [26,54], which is explained by the known natural tropism of porcine circoviruses for lymphoid tissues [55,56]. Furthermore, the studies previously carried out in wild boars from Spain focused on northeastern areas, where significant differences in the prevalence of other important wild boar diseases (e.g., TB) have been detected when compared to populations in mid-western Spain [57].

**Figure 2:**
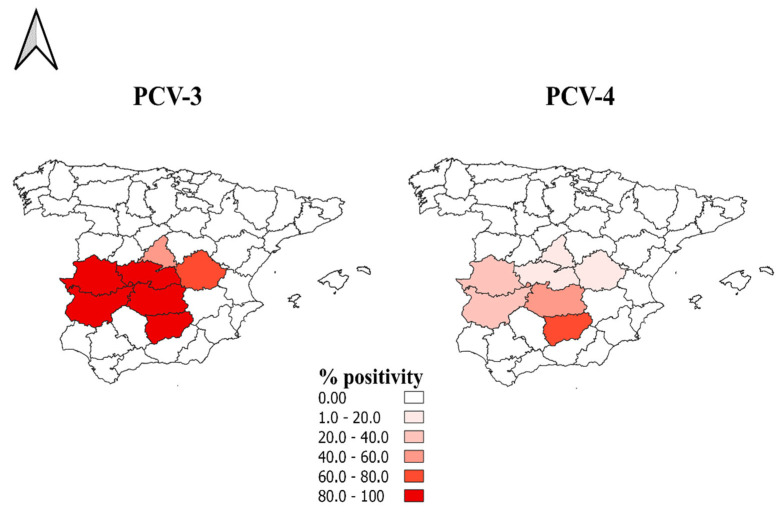
Percentage of positivity for PCV-3 and PCV-4 determined via PCR in wild boars sampled from different provinces of mid-western Spain.

Regarding PCV-4, the positivity rate was 33.7%. These data, previously reported by our research group in the first report in Spain [24], suggest a lesser distribution of this virus throughout wild boar populations from mid-western Spain compared to PCV-3. Although the animals included in this study were not used to estimate the prevalence of these pathogens, the presence of PCV-3 and PCV-4 in all of the areas under study, along with the small percentage of PCV-4 positivity, strongly suggests a similar spatial distribution but with a lower prevalence of PCV-4 in mid-western Spain (Figure 2). In addition, a moderately high rate of animals coinfected with PCV-3 and PCV-4 (30.12%) was detected; this contrasts with other research in which multiple types of PCVs were found, but PCV-3 and PCV-4 coinfection was not specifically described [32].

Individual features of animals, such as sex and age, do not seem to be factors that can influence the probability of infections with novel PCVs, since statistical differences between males/females and different age groups (young, subadult, and adult) were not found regarding PCV-3 and PCV-4 detection. The lack of relation between sex and PCV-3 infection is in agreement with similar previous studies, where the sex of wild boars did not influence the probability of becoming infected with PCV-2 or PCV-3 [11,37]. Regarding age, although there are no previous studies assessing the epidemiological effect of age on PCV-3 and PCV-4 infections, this absence of influence is in agreement with previous studies regarding PCV-2 infection in wild boars, where no significant statistical differences were found [37].

On the contrary, supplementary feeding seems to enhance the probability of PCV-4 infections in wild boars. This could be explained by the increase in direct contact that occurs when supplementing the feed of wild animals, since this management approach facilitates overcrowding and leads to more contact between animals than would occur in a natural feeding situation [58]. This is not the first time that a relationship has been found between intensive management and infections [37,59,60], even being related to endoparasite infections in wild boar populations [61].

Regarding the implications of PCV-3 and PCV-4 on the health status of infected wild boars, the results show little impact of these viruses on parameters related to reproductive status, lung pathology, or TB development. Although reproductive failure has been described in domestic pigs infected with PCV-3 and PCV-4 (abortion, mummification, below-average conception rates) [6,20], no implication of these novel PCVs on the reproductive parameters of wild boars has been detected. This aligns with previous studies that ruled out the effect of PCV-2 on the reproductive performance of wild boars [47], suggesting a high resistance of this species to reproductive adverse effects of circoviral infections. Similarly, lung lesions related to PCV-3 and PCV-4 infections have been described in domestic pigs [18,22]. However, infections with these viruses did not influence the lung pathological score obtained in the wild boars under study. No previous studies assess the effect of these recently described PCVs on lung pathology; however, experiments carried out on PCV-2 suggest that coinfections with *Mycoplasma hyopneumoniae* and/or other viral pathogens may be necessary to observe their effects on the development of lung lesions in wild boars [48]. In addition, some studies have shown that vasculitis and perivasculitis are characteristic histopathological lesions in PCV-3 infection [7,62], but such lesions were not associated with PCV-3-positive wild boars in this study. This may be due to the use of lung samples instead of lymph nodes in this research. Thus, it would be advisable to examine this type of lesion in lymph nodes in future research. Finally, the lack of impact of PCV-3 and PCV-4 on the development of a generalized TB pattern in wild boars also suggests a lack of influence of these pathogens on the immunological status of infected animals. This immunological implication has been reported in PCV2-infected animals [63], enhancing the probability of a more severe pattern of TB occurring in coinfected wild boars [64].

All of these results suggest that PCV-3 and PCV-4 infections do not represent a significant health threat to wild boars. However, the presence of high positivity rates for PCV-3 and moderate rates for PCV-4, despite the absence of clinical consequences, suggest that wild boars may act as reservoir for these viruses, posing a risk to domestic pigs, which have been demonstrated to be more susceptible to infection by these viruses [65]. This risk is higher in the areas examined in our study, where Iberian pigs are typically reared outdoors under extensive farming systems, and often have direct or indirect contacts with wild boars [66]. However, further epidemiological studies analyzing the phylogenetical relationships between the PCV-3 and PCV-4 strains detected in domestic and wild suids in this area will be necessary to elucidate the real potential of wild boars as reservoirs and disseminators of these pathogens.

## 5. Conclusions

PCV-3 and PCV-4 are widely distributed in wild boar populations in mid-western Spain, showing a similar spatial distribution, with a higher prevalence of PCV-3 than PCV-4. However, these pathogens do not appear to pose a risk to wild boar health, as no association with reproductive failure, lung damage, or TB development has been established.

## Figures and Tables

**Table 1:** Number of wild boars sampled in mid-western Spain and percentage of PCV-3 and PCV-4 positivity by province.

Province	Sampled Animals	% Positivity PCV-3	% Positivity PCV-4
Badajoz	45	97.7%	40%
Cáceres	32	84.4%	21.9%
Ciudad Real	24	83.3%	54.2%
Cuenca	9	77.8%	11.1%
Jaén	20	85%	70%
Madrid	10	60%	10%
Toledo	26	84.6%	7.7%
TOTAL	166	84.9%	33.7%

**Table 2:** Percentage of positivity for PCV-3 and PCV-4 and number of wild boars from mid-western Spain according to sex, age, and food supplementation. * significant differences = *p* value < 0.05.

	PCV-3			PCV-4		
Sex	Male	Female		Male	Female	
	42/53 (79.2%%)	91/104 (87.5%)		21/53 (39.6%)	34/104 (32.7%)	
Age	Young	Subadult	Adult	Young	Subadult	Adult
	14/19 (73.7%)	15/20 (75%)	97/111 (87.4%)	5/19 (26.3%)	10/20 (50%)	37/111 (33.3%)
Feed supplementation	Yes	No		Yes	No	
	117/133 (88%)	24/32 (75%)		51/133 (38.3%) *	5/32 (15.6%)	

**Table 3:** Mean body condition and reproductive parameters (number of breeding females, mean potential fertility, and mean intrauterine mortality observed in PCV-3-/PCV-4-positive and -negative wild boars from mid-western Spain. * significant differences = *p* value < 0.05.

	PCV-3		PCV-4	
	Positive	Negative	Positive	Negative
Mean body condition (mm)	17.6 *	14.3	16.5	17.3
Breeding females (n)	46/74 (86.8%)	7/10 (15.1%)	15/37 (30.2%)	38/56 (22.6%)
Mean potential fertility (piglets)	3.7	2.8	3.4	3.7
Mean intrauterine mortality (%)	14.64%	36.67%	16.8%	16.9%

**Table 4:** Mean values obtained for five histopathological parameters assessed to evaluate lung damage and total lung damage score in wild boars from mid-western Spain.

	PCV-3		PCV-4	
	Positive	Negative	Positive	Negative
Interstitial inflammatory infiltration (0−6)	0.92	1	0.95	0.92
Alveolar exudates (0−6)	0.28	0	0.26	0.23
BALT hyperplasia (0−6)	1.34	1.75	1.37	1.42
Peribronchial inflammatory infiltration (0−6)	0.41	0.35	0.3	0.45
Epithelial necrosis (0−6)	0	0.05	0	0
Total lung damage score (0−30)	3.4	4.1	3.6	3.5

## Data Availability

The data that support the findings of this study are available from the corresponding author upon reasonable request.

## Supplementary Materials

The following supporting information can be downloaded at https://www.mdpi.com/article/10.3390/ani15040523/s1: Table S1: Dataset used to support results obtained in this study. Table S2: Histological scores obtained during pathological lung examination.

## Abbreviations

The following abbreviations are used in this manuscript:

PCV-3	porcine circovirus 3
PCV-4	porcine circovirus 4
PDNS	Porcine Dermatitis and Nephropathy Syndrome
